# Functional roles of ADP-ribosylation writers, readers and erasers

**DOI:** 10.3389/fcell.2022.941356

**Published:** 2022-08-11

**Authors:** Ping Li, Yushuang Lei, Jia Qi, Wanqin Liu, Kai Yao

**Affiliations:** Institute of Visual Neuroscience and Stem Cell Engineering, College of Life Sciences and Health, Wuhan University of Science and Technology, Wuhan, China

**Keywords:** ADP-ribosylation, ARTs, PAR recognition domain, de-ADP-ribosylating enzymes, post-translational modification

## Abstract

ADP-ribosylation is a reversible post-translational modification (PTM) tightly regulated by the dynamic interplay between its writers, readers and erasers. As an intricate and versatile PTM, ADP-ribosylation plays critical roles in various physiological and pathological processes. In this review, we discuss the major players involved in the ADP-ribosylation cycle, which may facilitate the investigation of the ADP-ribosylation function and contribute to the understanding and treatment of ADP-ribosylation associated disease.

## 1 Introduction

**FIGURE 1:**
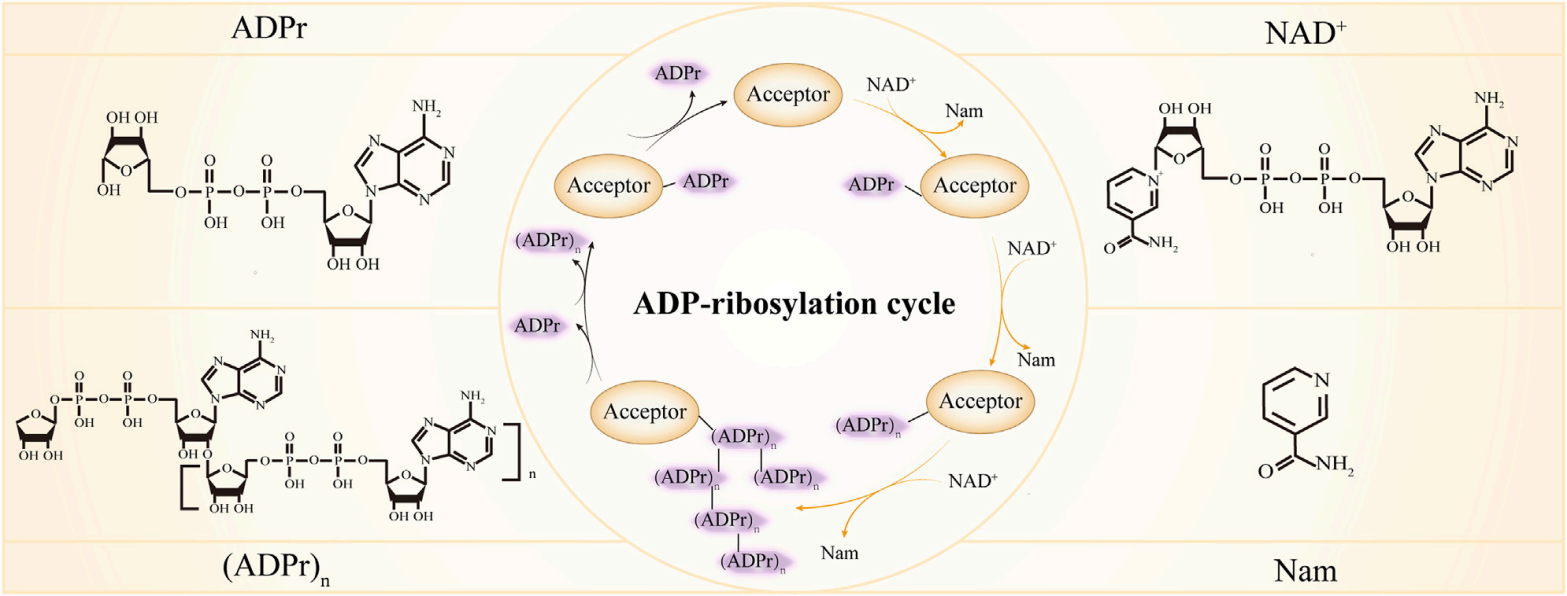
ADP-ribosylation cycle. ADPr, ADP-ribose; NAD^+^, Nicotinamide adenine dinucleotide; Nam, nicotinamide. In the center inner circle, the reactions indicated by the yellow arrows are catalyzed by ADP-ribosylation writers, the reactions indicated by the black arrows are catalyzed by ADP-ribosylation erasers and different forms of ADP-ribose are recognized by ADP-ribosylation readers.

ADP-ribosylation is the enzymatic reaction whereby ADP-ribose monomer (MAR) or polymer (PAR) are covalently attached to the substrates. As an intricate and versatile post-translational modification (PTM), ADP-ribosylation is receiving growing attention due to its involvement in a broad range of biological processes like DNA damage repair, transcription, chromatin regulation, cell cycle control, cell senescence, apoptosis, and necrosis (Chou et al., 2006; Messner et al., 2010; Yang L. et al., 2017; Gupte et al., 2017; Chen et al., 2018; Luscher et al., 2018; Zhang L. et al., 2020; Menzel et al., 2021; Fu et al., 2022). Dysregulation of ADP-ribosylation has been linked to numerous diseases, such as neurodegeneration, cancer and inflammation (Aguiar et al., 2000; Mehrotra et al., 2013; Butepage et al., 2015; Palazzo et al., 2019; Challa et al., 2021; Rosado and Pioli, 2021; Vermehren-Schmaedick et al., 2021), highlighting the importance of maintaining ADP-ribosylation balance. A repertoire of proteins has been shown to work in concert to fine-tune ADP-ribosylation signal and tightly regulate the ADP-ribosylation process (the ADP-ribosylation cycle is shown in Figure 1). According to their function, the proteins can be described as ADP-ribose writers that add MAR or PAR, readers that disseminate the information carried by ADP-ribose, and erasers that remove the MAR or PAR modifications (Cohen and Chang, 2018; O'Sullivan et al., 2019). Here we summarize our current understanding of the major players in the ADP-ribosylation regulation and discuss their role in various aspects of the cellular processes.

## 2 Writers of ADP-ribosylation

The writers of ADP-ribosylation cleave nicotinamide adenine dinucleotide (NAD^+^) to nicotinamide (Nam) and ADP-ribose, and successively transfer the ADP-ribose to various targets. Proteins are the best-characterized targets of ADP-ribosylation writers and many amino acid residues including glutamic, aspartic acids (Zhang et al., 2013), serines (Palazzo et al., 2018), threonines (Yan et al., 2020), arginine (Vandekerckhove et al., 1987; Koch-Nolte et al., 1996) and cysteines (Vyas et al., 2014) can serve as acceptors for ADP-ribosylation. Additionally, recent studies have shown that both DNA (Takamura-Enya et al., 2001; Lyons et al., 2016; Belousova and Lavrik, 2018; Groslambert et al., 2021) and RNA (Munir et al., 2018; Munnur et al., 2019; Groslambert et al., 2021) can be ADP-ribosylated. ADP-ribosyltransferases (ARTs) are the main writers of ADP-ribosylation. The ARTs that transfer only one ADP-ribose unit and produce MARylated substrates are termed mono-ADP-ribosyltransferases (mono-ARTs), and this modification is called MARylation, while ARTs that synthesize and transfer the linear or branched ADP-ribose polymer, forming PARylated substrates, are named poly- ADP-ribosyltransferases (poly-ARTs), and this reaction is called PARylation. There are 23 members in the ADP-ribosyltransferase superfamily (El-Gebali et al., 2019; Wyzewski et al., 2021). Their characteristic feature is the ART fold which enables the binding of NAD^+^ and catalyzes the transfer of ADP-ribose. Based on three conserved amino acids in the ART fold that are critical for its proper function, ARTs are classified into three clades (the H-Y-[EDQ] clade, the R-[ST]-E clade, and the H-H-h clade). While ARTs containing H-Y-[EDQ] motif were first described for diphtheria toxin of *Corynebacterium diphtheria* and thus were designated as ARTDs (ADP-ribosyltransferase diphtheria-toxin like), ARTs containing R-[ST]-E motif were related to cholera toxin from *Vibrio cholerae* and as such were called ARTCs (ADP-ribosyltransferase cholera-toxin like) (Hottiger et al., 2010; Luscher et al., 2021). The focus here will be on the ARTDs and ARTCs and we will discuss studies about ARTDs and ARTCs in more detail in the following sections.

### 2.1 ARTD family

**FIGURE 2:**
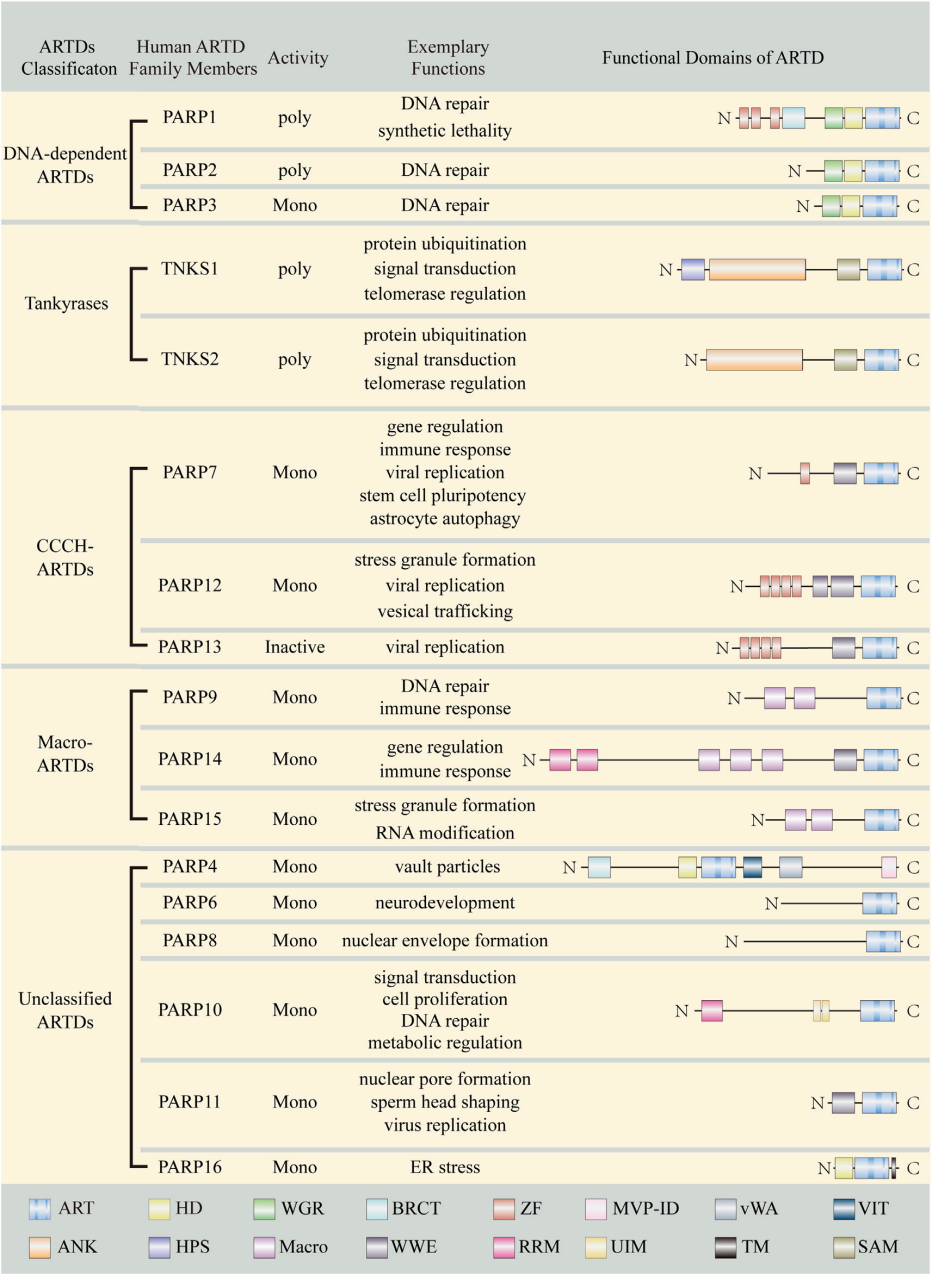
Summary of ARTD family members. Important domains of ARTD family members are indicated with different color boxes. ART, ADP-ribosyltransferase domain; HD, helical domain; WGR, Trp-Gly-Arg motif; BRCT, BRCA1 C-terminus-like motif; ZF, Zinc finger; MVP-ID, M-vault particle interaction domain; vWA, von Willebrand factorm type A; VIT, vault protein inter-alpha-trypsin; SAM, sterile alpha motif; ANK, Ankyrin repeats; HPS, Histidine-proline-serine region; Macro, Macrodomain; WWE, Tryptophan (W)-Tryptophan (W)-Glutamate (E); RRM, RNA recognition motif; UIM, ubiquitin interaction motif; TM, transmembrane motif.

The majority of mammalian ARTs belong to the ARTD subfamily (Hottiger et al., 2010; Luscher et al., 2021). There are 17 ARTDs in humans (Schreiber et al., 2006; Cohen and Chang, 2018) (Figure 2). These proteins were previously also called poly ADP-ribose polymerases (PARPs). Since most ARTDs don’t synthesize PAR, it has been proposed that ADP-ribosyltransferase should be adopted as a unified nomenclature and the term PARP is just a name standing for the different ART members (Hottiger et al., 2010; Luscher et al., 2021). Among ARTDs, only four possess PARylation activity, namely: PARP1, PARP2, TNKS1, and TNKS2. The remaining members perform MARylation except for PARP13, which appears to be catalytically inactive (Kleine et al., 2008; Vyas et al., 2014; Poltronieri et al., 2021). Of note, although PARP9 is enzymatically inactive in isolation, it displays MARylation activity when it forms a complex with ubiquitin E3 ligase DTX3L (Aguiar et al., 2005; Yang C. et al., 2017), thus we categorize PARP9 as mono-ART. Based on the domain architectures and functions, ARTDs can be broadly classified into five categories: DNA-dependent ARTDs, Tankyrases, Cys-Cys-Cys-His (CCCH) ARTDs, macro-ARTDs and other unclassified ARTDs.

#### 2.1.1 DNA-dependent ARTDs

DNA-dependent ARTDs comprising PARP1, PARP2, and PARP3, are characterized by their DNA-dependent enzymatic activity (Yamanaka et al., 1988; Langelier et al., 2012; Langelier et al., 2014; van Beek et al., 2021). PARP1 is the founding member of the ARTD family and encompasses three functional domains: an N-terminal DNA-binding domain that contains three zinc finger motifs (Langelier et al., 2010; Eustermann et al., 2011; Langelier et al., 2011), a central automodification domain that has a BRCA1 C-terminal (BRCT) domain, a helical domain (HD) and a Trp-Gly-Arg (WGR) domain (Langelier et al., 2012) and a C-terminal catalytic domain that is responsible for ADP-ribosylation (Kameshita et al., 1984; Kurosaki et al., 1987). Following genotoxic insult, PARP1 is rapidly recruited to the broken DNA ends via its DNA-binding domain and is catalytically activated in its catalytic domain (Langelier et al., 2012). Activated PARP1 PARylates itself (auto-PARylation), histones, and other proteins (Ogata et al., 1981; Huletsky et al., 1989; D'Amours et al., 1999; Schreiber et al., 2006; Dabin et al., 2016; Zhang and Li, 2019), and further recruits DNA repair factors to fix the lesions (Ahel et al., 2009; Chou et al., 2010; Ying et al., 2016; Zhang and Li, 2019). PARP2 and PARP3 share a similar C-terminal region (catalytic domain, HD and WGR domain) with PARP1. While PARP3 catalyzes MARylation, PARP1, and PARP2 catalyze PARylation. Moreover, PARP1 displays higher processivity than PARP2, with PARP1 accounting for approximately 90% of PAR generation and PARP2 contributing to the remainder (Ame et al., 1999). The structure of PARP1-3 diverges at their N-terminus. PARP2 and PARP3 lack the zinc finger motifs and BRCT domain, but they can still bind to DNA with WGR domain and play an essential role in DNA repair (Eustermann et al., 2011; Langelier et al., 2014; Riccio et al., 2016; Chen et al., 2018; Obaji et al., 2021). PARP1-3 act as DNA damage sensors and can recognize different types of DNA damage. Specifically, PARP1 has a broad substrate range and responds to DNA lesions like single-and double-strand breaks, DNA-crosslinks and stalled replication forks (Leppard et al., 2003; Lonskaya et al., 2005; Eustermann et al., 2011; Langelier et al., 2011; De Vos et al., 2012; Ronson et al., 2018). PARP2 recognizes DNA gaps and flaps (Sukhanova et al., 2016; Talhaoui et al., 2016; Obaji et al., 2018), whereas PARP3 prefers mononucleosomes containing nicked DNA (Grundy et al., 2016) and is implicated in single-strand DNA breaks (Langelier et al., 2014; Grundy et al., 2016). Moreover, both PARP2 and PARP3 are preferentially activated by 5’ phosphorylated DNA breaks, whereas no clear preference for any type of DNA breaks is observed in PARP1 (Langelier et al., 2014). Even in the absence of DNA, PARP1 can be activated by PARP3 (Loseva et al., 2010). Consistent with their critical role in DNA damage response, *parp1* knockout mice and cells show increased sensitivity to DNA strand interruptions (de Murcia et al., 1997; Shibata et al., 2005). Like PARP1, *parp2* knockout mice also show hypersensitivity to sublethal γ-irradiation (Farres et al., 2013). Nevertheless, PARP1, but not PARP2, is required for protecting cells from spontaneous toxic recombinant lesions and co-depleting PARP1 rather than PARP2 with BRCA2 (important for homologous recombination) leads to synthetic lethality in human cells (Bryant et al., 2005). Synthetic lethality refers to a situation where the concomitant alteration of two or more genes causes cell death, whereas alteration of either gene alone is compatible with cell viability. The synthetic lethality of PARP1 and BRCA1/2 seen in tumor cells (Bryant et al., 2005; Farmer et al., 2005) has led to intensive investigations of developing PARP inhibitors to treat cancers with deficiencies in BRCA or other DNA repair genes such as p53 (Williamson et al., 2012), MRE11 (Vilar et al., 2011), PTEN (Mendes-Pereira et al., 2009), MTA2 (Shi et al., 2021) and so on (Lord et al., 2008; Tutt et al., 2010; Cardnell et al., 2013; Kaufman et al., 2015; de Bono et al., 2017; Scott, 2017; Hoy, 2018; Litton et al., 2018). Currently, four PARP inhibitors (olaparib, rucaparib, niraparib and talazoparib) have been approved by FDA for cancer therapy (https://www.fda.gov/drugs) and many others are under clinical trials (NCT01827384, NCT01605162, and veliparib) (https://clinicaltrials.gov/ct2/home) due to their synthetic lethality mechanism. Notably, although veliparib is still in phase 3 clinical trials, it has been approved under an orphan designation for the treatment of ovarian cancer (https://www.ema.europa.eu/en/medicines/human/orphan-designations/eu310830).

#### 2.1.2 Tankyrases

TNKS1 and TNKS2 constitute the Tankyrases group. They share an 81% nucleotide homology and 85% amino acid identity (Kaminker et al., 2001) and have a similar structural organization (Kaminker et al., 2001). Both TNKS1 and TNKS2 contain a C-terminal catalytic ART domain, a middle SAM (sterile alpha module) domain and an N-terminal ANK (ankyrin) repeat domain (Smith et al., 1998; Kaminker et al., 2001). Except for these common domains, TNKS1 additionally has a unique histidine-proline-serine rich (HPS) motif at the N-terminal region, but the function of this motif is still unknown (Kaminker et al., 2001; Lyons et al., 2001). The unique domain organization, particularly the SAM domain and the ANK repeat domain distinguishes Tankyrases from other members of the ARTDs family. While the SAM domain of Tankyrases mediates their homo- or hetero-oligomers formation and regulates their catalytic activity (De Rycker and Price, 2004; Fan et al., 2018), the ANK domain of Tankyrases serves as a protein interaction interface and is responsible for substrates recognization (Eisemann et al., 2016). Most of characterized Tankyrase substrates contain canonical Tankyrase binding motif (TBM) with consensus sequence RXXPXG (Sbodio and Chi, 2002; Guettler et al., 2011) or non-canonical TBM with a sequence of RXXAXG or RXXXXG (Croy et al., 2016; Li et al., 2017; Tripathi and Smith, 2017). A number of Tankyrase binding partners have been identified, including 3BP2 (Guettler et al., 2011), TRF1 (Smith et al., 1998), Axin1/2 (Callow et al., 2011), PTEN (Li N. et al., 2015) and so on. The recognition of substrates by Tankyrases triggers various biological responses such as altering protein stability (Callow et al., 2011; Guettler et al., 2011; Li N. et al., 2015), modifying catalytic activity (Bisht et al., 2012)and changing protein-protein interaction (Li et al., 2019; Zheng et al., 2019). Among them, one of the most studied Tankyrases effect is controlling the substrate stability with PAR-dependent ubiquitination (PARdU). In the PARdU process, Tankyrase binds to and PARylates the target proteins, and then E3-ubiquitin ligases RNF146 recognizes and adds Lys-48 linked poly-ubiquitin chains on the PARylated proteins, which promotes their proteasomal degradation (Zhang et al., 2011; Vivelo et al., 2019). Given the diverse substrates and complicated biological responses, Tankyrases impinge on a broad range of cellular processes including telomere elongation (Smith and de Lange, 2000), DNA repair (Yang L. et al., 2017), mitosis (Canudas et al., 2007), signal transduction (Callow et al., 2011; Zhang et al., 2011; Verma et al., 2021; Mashimo et al., 2022) and metabolism regulation (Zhong et al., 2016; Li P. et al., 2018; Li et al., 2019).

#### 2.1.3 CCCH-ARTDs

The CCCH-ARTDs subfamily is composed of three members: PARP7, PARP12, PARP13, and have common WWE (Tryptophan-Tryptophan-Glutamate) domains that recognize ADP-ribose (discussed in detail in the “Readers” section) and Cys-Cys-Cys-His zinc fingers that interact with cytoplasmic RNAs or specific viral RNAs (Guo et al., 2004; Guo et al., 2007; Liu and Yu, 2015). PARP7 is a mono-ART, which also refers to 2,3,7,8-tetrachlorodibenzo-p-dioxin (TCDD)-inducible PARP (TiPARP) because it was first identified as a TCDD-induced target gene of aryl hydrocarbon receptor (AHR) (Ma et al., 2001). AHR is a ligand-activated transcription factor. PARP7 plays an important role in transcription regulation and functions as part of a negative feedback regulator to restrain AHR transcription activity by MARylating it (MacPherson et al., 2013). Besides, PARP7 can negatively regulate other transcription factors such as HIF-1α and estrogen receptor. Mechanistically, PARP7 promotes the ubiquitination and proteasomal degradation of HIF-1α and estrogen receptor by recruiting them and E3 ubiquitin ligase HUWE1 to nuclear condensates in an ADP ribosylation-dependent manner (Zhang L. et al., 2020). Moreover, PARP7 can MARylate agonist-bound androgen receptor (AR) on its N-terminal cysteine residues and regulate AR-dependent gene transcription (Kamata et al., 2021; Yang et al., 2021). In addition to the regulation of transcription, PARP7 appears to have both anti- and pro-viral activities against different viruses. During SINV (Sindbis virus) infection, PARP7 exhibits antiviral activity by binding to and promoting the degradation of SINV RNA and abrogation of PARP7 enhances SINV replication (Kozaki et al., 2017). Whereas during vesicular stomatitis virus (VSV), Newcastle disease virus (NDV), influenza virus (FluV) and encephalomyocarditis virus (EMCV) infection, PARP7 shows pro-viral activity and negatively regulates IFN-I (type I interferon) antiviral response by MARylating TANK binding kinase (TBK1). PARP7 deficiency restricts the replication of these viruses (Yamada et al., 2016). Consistent with this notion, a separate study demonstrates that PARP7 knockdown reduces MHV (mouse hepatitis virus) replication and increased IFN-I expression (Grunewald et al., 2020). IFNs are pleiotropic cytokines that not only exhibit antiviral effects but also have antitumor properties. A recent study shows that PARP7 expression is upregulated in cancers with downregulated IFN signaling where it acts as a tumor cell brake in IFN-I response. Inhibition of PARP7 by a selective inhibitor, RBN-2397, restores IFN-I response and contributes to tumor regression by inhibiting cell proliferation and activating the immune system (Gozgit et al., 2021). Currently, RBN-2397 has entered clinical testing for the treatment of solid tumors because of its promising antitumor activity (Falchook et al., 2021). Moreover, in ovarian cancer cells, PARP7 mediates MARylation of α-tubulin and promotes microtubule instability, which facilitates cell growth and motility. PARP7 knockdown leads to a reduction of growth, migration and invasion of ovarian cancer cells (Palavalli Parsons et al., 2021), indicating inhibition of PARP7 may have a positive therapeutic effect on ovarian cancer. However, the opposite role of PARP7 in cancer development has been reported. In the colon xenografts, PARP7 overexpression inhibits while PARP7 knockdown promotes tumor growth. The tumor-promoting effect of PARP7 knockdown is also observed in the breast cancer xenografts and lower PARP7 expression is significantly correlated with poor prognosis of patients with breast cancer (Zhang L. et al., 2020). Overall, these inconsistent findings suggest that PARP7 may have tumor cell-specific effects. The functions of PARP7 are much more than those mentioned above. It is also involved stem cell pluripotency (Roper et al., 2014) and astrocyte autophagy (Han et al., 2018). PARP12 is a Golgi-localized mono-ART. The best-characterized function of PARP12 is its role in virus infection. Under stress challenge, PARP12 can translocate from the Golgi complex to stress granules and lead to disassembly of the Golgi complex, which inhibits the anterograde-membrane traffic (a route that many viruses rely on for particle assembly) (Catara et al., 2017). In addition to disrupting the virus particle assembly, PARP12 can also suppress virus infection by MARylating viral proteins and causing their degradation (Li L. et al., 2018). PARP13, which is catalytically inactive, has antiviral activities and affects viral replication independently of ADP-ribosylation (Gao et al., 2002; Guo et al., 2007; Li M. et al., 2015; Chiu et al., 2018; Fehr et al., 2020; Luo et al., 2020). The antiviral effects of PARP13 are multi-faceted. On the one hand, it stimulates the degradation of viral mRNA by directly binding to viral RNA and recruiting the RNA-processing exosome complex (Guo et al., 2007). On the other hand, it suppresses the translation of viral mRNA by disrupting the interaction between translational initiation factors eIF4A and eIF4G (Zhu et al., 2012; Fehr et al., 2020).

#### 2.1.4 Macro-ARTDs

Macro-ARTDs consisting of PARP9, PARP14, and PARP15 are characterized by the tandem macrodomains in their N-terminus. Macrodomains are responsible for ADPr binding. Its characteristics will be discussed in detail in the “Readers” section. PARP9 has two macrodomains and is initially thought to be inactive due to lack of automodification activity in its catalytic domain (Vyas et al., 2014). Later study shows that PARP9, in a complex with DTX3L, can MARylate the C-terminus of ubiquitin on Gly76. In the context of PARP9/DTX3L heterodimer, the catalytic domain of PARP9 is required for ubiquitin ADP-ribosylation (Yang C. et al., 2017). PARP9/DTX3L complex has dual activities (E3 and ADP-ribosyltransferase activities) (Takeyama et al., 2003; Yang C. et al., 2017; Yang et al., 2021) and both activities are observed in ubiquitin processing (Yang C. et al., 2017). However, DTX3L is reported to can ADP-ribosylate ubiquitin on its own (Chatrin et al., 2020) and the presence of PARP9 can enhance both the E3 and ADP-ribosyltransferase activities of DTX3L (Ashok et al., 2022), so the catalytic activity of PARP9 needs further investigation. PARP9/DTX3L contributes to DNA repair via non-homologous end joining (NHEJ) and knockdown down of either gene enhances cell sensitivity to DNA damage agents (Yang C. et al., 2017). In addition to DNA repair, PARP9 in itself or complex with DTX3L shows pro-tumorigenic functions via regulating IFN signaling. In metastatic prostate cancer cells, PARP9 and DTX3L inhibit the expression of tumor suppressor IRF1, thereby counteracting the IFNγ-dependent host immune response against tumor cells (Bachmann et al., 2014). In diffuse large B-cell lymphomas, PARP9 functions as a co-repressor of the IFNγ-STAT1-IRF1 axis to inhibit apoptosis and as a co-activator of the IFNγ-STAT1-BCL6 axis to promote cell proliferation (Camicia et al., 2013). Outside of the context of cancer, PARP9 has been identified as a noncanonical sensor for RNA viruses that initiates and amplifies IFN-I production. PARP9 deficient mice are more sensitive to RNA virus infections due to IFN-I production impairment (Xing et al., 2021). PARP14 is a mono-ART with three macrodomains. Specifically, the Macro2 domain of PARP14 has been shown to recognize MARylation. Therefore, it is both writer and reader of MARylation (Forst et al., 2013). PARP14 has been identified to involve in multiple cellular processes, such as STAT6 activation (Mehrotra et al., 2011), B-cell differentiation (Iansante et al., 2015), signal transduction (Iansante et al., 2015; Yao et al., 2019), genomic stability (Nicolae et al., 2015) and RNA stability (Iqbal et al., 2014; Carter-O'Connell et al., 2018). As a transcriptional coactivator for STAT6, PARP14 has been regarded as a potential anti-cancer and anti-inflammatory target, which has been reviewed in detail elsewhere (Schweiker et al., 2018; Qin et al., 2019; Tauber et al., 2020). Moreover, recent studies have demonstrated that PARP14 has a significant antiviral role (Eckei et al., 2017; Caprara et al., 2018) and it is proposed that PARP14 may help to activate the host defense against the SARS-CoV-2 and counteract the immune imbalance caused by SARS-CoV-2 (Tauber et al., 2021). Compared with PARP14, less is known about PARP15. PARP15 is a mono-ART with two macrodomains that may bind to either mono-ADP-ribose or poly-ADP-ribose. Currently, little is known about the PARP15 function. Yet studies have shown that PARP15 is involved in stress granules formation (Leung et al., 2011) and is inactive in head and neck squamous cell carcinoma (Guerrero-Preston et al., 2014). Moreover, Munnur et al. (2019) reported that PARP15 can MARylate phosphorylated ends of RNA.

#### 2.1.5 Unclassified ARTDs

The remaining ARTDs share no domains in common and do not fit into any of the previous categorizations, thus are referred to as unclassified ARTDs. These ARTDs include PARP4, PARP6, PARP8, PARP10, PARP11, and PARP16. PARP4, also known as PARP4 or vPARP, was originally discovered in vault particles (Kickhoefer et al., 1999). The presence of PARP4 in vaults that are responsible for drug resistance and nucleocytoplasmic transport suggests a relevant PARP4 ability, which however still needs more experimental proof. The function of PARP4 is not fully understood at present. Although PARP4 has been shown to bind to TEP1 (a telomerase subunit), it is dispensable for telomerase regulation (Liu et al., 2004). Moreover, early research indicated that PARP4-deficient mice had no obvious developmental defect (Liu et al., 2004), and subsequent work declared that PARP4-deficient mice were prone to carcinogen-induced tumors (Raval-Fernandes et al., 2005), suggesting that PARP4 may function as a tumor suppressor. Consistently, whole-exome sequencing in patients with primary thyroid and breast cancer shows that the PARP4 mutation frequency is significantly higher than that in control individuals (Ikeda et al., 2016). Whereas functional analysis indicates that PARP4 knockout inhibits the proliferation and colony formation of breast cancer cells (Prawira et al., 2019), thus further investigations are warranted to clarify the role of PARP4 in cancer. The catalytic activity of PARP4 also remains elusive. PARP4 shows MARylation activity by itself (*in vitro*), but the PARP4-containing vault complexes exhibit PARylation activity (Kickhoefer, et al., 1999), which could be due to PARP4 in its native protein-complex form or other co-purifying ARTDs. PARP6 is a neuronally enriched mono-ART and its expression peaks during a critical period of hippocampal dendrite morphogenesis. Overexpression of PARP6 promotes, whereas knockdown of PARP6 suppresses dendritic growth and branching in primary rat hippocampal neurons (Huang et al., 2016), suggesting that PARP6 is a positive regulator of hippocampal dendrite morphogenesis during neurodevelopment. Consistently, several neurodevelopmental disorders are observed in four patients with PARP6 mutation in the catalytic domain (Vermehren-Schmaedick et al., 2021). Apart from its role in neuron development, PARP6 is involved in cancer progression. PARP6 expression is a biomarker for good prognosis of colorectal cancers (CRC) (Tuncel et al., 2012) and the increased methylation of PARP6 is positively correlated with poor prognosis of hepatoblastoma (Honda et al., 2016), indicating the tumor suppressor role of PARP6. Qi et al. (2016) report that the tumor suppressive function of PARP6 in CRC is elicited by downregulating the expression of survivin, an anti-apoptosis gene. However, Wang et al. (2017) report that PARP6 knockdown only slightly inhibits survivin expression and survivin is overexpressed in CRC. Sun et al. (2018) even observe a positive correlation between PARP6 and survivin expression in gastric cancer and the authors proposed that PARP6 acts as an oncogene via activating the survivin pathway in gastric cancer. In addition to regulating survivin expression, Wang et al. (2018) demonstrate that PARP6 maintains centrosome integrity by ADP-ribosylating checkpoint kinase 1 (CHK1) in breast cancer cells and targeting PARP6 with small molecule inhibitor AZ0108 induces multipolar spindle formation and inhibits tumor growth in breast cancer models (Wang et al., 2018). Nevertheless, Howard et al. show that PARPYnD can inhibit PARP6 *in vitro* and induce multipolar spindle formation like AZ0108, but it fails to bind to PARP6 in the intact cell, indicating PARP6 inhibition may not be the only mechanism whereby these molecules exert antitumor effect (Howard et al., 2020). PARP8 has no defined domains except for the catalytic domain that possesses MARylation activity (Vyas et al., 2014). PARP8 is primarily localized to the nuclear envelope during interphase and at the spindle poles during mitosis. PARP8 knockdown results in abnormal, bilobed nuclei and leads to cell morphology defects together with a pronounced decrease in cell viability (Vyas et al., 2013). To date, the functions of PARP8 are poorly understood, yet it is observed overexpressed in CRC, implying PARP8 might act as an oncogene for CRC (Yu and Zhu, 2022). PARP10 is the founding member of mono-ARTs and mainly localizes to the cytosol but appears to shuttle between cytoplasm and nucleus. In the nucleus, PARP10 has been found to bind to the nuclear oncogenic transcription factor, c-MYC, and suppress its transforming activity (Yu et al., 2005). Additionally, nucleolar PARP10 has been shown to function in cell proliferation and is required for G1 to S cell cycle transition (Chou et al., 2006). PCNA is a major coordinator of cell cycle and DNA repair (Nichols and Sancar, 1992; Gulbis et al., 1996; Chen et al., 2013). PARP10 has been shown to bind to ubiquitinated PCNA and directly participate in DNA repair (Nicolae et al., 2014). In the cytosol, PARP10 participates in various cellular processes by performing MARylation on its target proteins. A wide range of cytosolic PARP10 substrates have been identified, such as GSK3β and NEMO. While the MARylation of GSK3β by PARP10 inhibits its kinase activity, suggesting the possible correlation between PARP10 with other signal pathways (Feijs et al., 2013b), the MARylation of NEMO prevents its K63-linked poly-ubiquitination, providing evidence for cross-talk between MARylation and poly-ubiquitination (Verheugd et al., 2013). Recent works have provided novel insight into the PARP10 function. Zhao et al. illustrated that PARP10 suppressed tumor metastasis by MARylating Aurora A, a serine/threonine kinase, and inhibiting its kinase activity (Zhao et al., 2018) and Tian et al. (2020) found that PARP10 inhibited the oncogenic function of PLK1 by MARylating it, whereas Schleicher et al. proved that PARP10 promoted tumorigenesis by alleviating replication stress (Schleicher et al., 2018). Additionally, Marton et al. (2018) suggested that PARP10 regulated metabolic processes by modulating mitochondrial function. PARP11 is a small mono-ART with only a single N-terminal WWE domain and a C-terminal catalytic domain, which endow its proper function. PARP11 prefers to reside in the nuclear envelope and is required for nuclear envelope stability. The absence of either WWE domain or catalytic domain abolishes its subcellular localization preference and the knockout of *parp11* in mice leads to teratozoospermia with abnormal head shapes because of nuclear envelope defect (Meyer-Ficca et al., 2015). Apart from the role in sperm head shaping, PARP11 is involved in viral infection. It exhibits pro-viral function during VSV (vesicular stomatitis virus) and HSV-1 (herpes simplex virus-1) infection. In this process, PARP11 inhibits the interferon antiviral response by MARylating the ubiquitin E3 ligase β-TrCP (β-transducin repeat-containing protein) and triggering the ubiquitination and degradation of IFNAR1 (IFNα/β receptor subunit 1) (Guo et al., 2019). Nevertheless, PARP11 shows an anti-viral effect in the presence of PARP12 during ZIKV (Zika virus) infection, which is independent of its catalytic domain. Instead, it suppresses ZIKV replication by interacting with PARP12 via its WWE domain and promotes PARP12-mediated ZIKV protein degradation (Li et al., 2021). PARP16 is the only mono-ART with a C-terminal transmembrane domain. It localizes to either the nuclear envelope or the endoplasmic reticulum (ER) (Butepage et al., 2015). In line with its subcellular localization, PARP16 regulates the unfolded protein response (UPR) of the ER by modifying IRE1α (inositol requiring enzyme 1) and PERK (protein kinase RNA-like ER kinase) (Jwa and Chang, 2012), and influences nuclear-cytoplasmic transport by interacting with and ADP-ribosylating karyopherin-β1 (Di Paola et al., 2012).

### 2.2 ARTC family

The ARTC family members are characterized by their catalytic domain that is structurally related to cholera toxin (Glowacki et al., 2002; Luscher et al., 2021). There are four members of the ARTC family (ARTC1, ARTC3, ARTC4, and ARTC5) expressed in humans and six in mice (Artc1, Artc2.1, Artc2.2, Artc3, Artc4, and Artc5) (Glowacki et al., 2002). The human gene encoding ARTC2 is non-functional due to the premature exonic stop codons, whereas the mouse *Artc2* encodes two gene products, Artc2.1 and Artc2.2 (Prochazka et al., 1991; Haag et al., 1994). Both Artc2.1 and Artc2.2 MARylate their protein substrates on arginine residues. Artc1 and Artc5 also show Arg-specific MARylation activity, yet Artc3 and Artc4 have no catalytic activity (Koch-Nolte et al., 2008; Di Girolamo and Fabrizio, 2019).

Artc1 and Artc2 are the most studied members of the ARTC family. Artc1 was originally identified from skeletal muscle microsomal membranes (Peterson et al., 1990). An early study demonstrated that Artc1 ADP-ribosylated Integrin alpha7 in the skeletal muscle cells and was suggested to be involved in myogenesis (Zolkiewska and Moss, 1993). Consistent with this view, a subsequent study reported that Artc1-deficient mice exhibited signs of muscle weakness and many Artc1-MARylated proteins were identified in skeletal muscle cells and tissues (Leutert et al., 2018), further indicating the important role of Artc1 in muscle development and muscle function. Additional Artc1 functions have also been found later. For example, Artc1 has been shown to participate in the airway inflammatory response by MARylating human neutrophil peptide 1(HNP-1), a well-known antimicrobial peptide. As ADP-ribosylated HNP-1 is observed in bronchoalveolar lavage fluids (BALF) of the patient with asthma and pulmonary fibrosis (Paone et al., 2002; Paone et al., 2006), it is proposed that Artc1 may be a potential therapeutic target for these diseases. Furthermore, although the majority of mammalian ARTCs are bound to the cell membrane with a glycosylphosphatidylinositol (GPI)-anchor (Glowacki et al., 2001; Di Girolamo and Fabrizio, 2019), Artc1 has been shown to localize not only to the cell periphery but also to the endoplasmic reticulum (ER), where it MARylates the GRP78/BiP, a luminal ER molecular chaperone, causing its inactivation, and regulating the ER stress (Fabrizio et al., 2015).

Artc2 is expressed in two allelic forms, Artc2.1 and Artc2.2. Artc2.1 is predominantly expressed on macrophages, dendritic cells, T cells, as well as microglia, whereas Artc2.2 mainly presents on T cells (Glowacki et al., 2002; Hong et al., 2009). Artc2 participates in a large number of immune response modulation mechanisms. In the bone marrow-derived macrophages, Artc2.1 is upregulated in response to proinflammatory mediators (Hong et al., 2007). In microglia, the expression of Artc2.1 is strongly induced by IFNβ, which is followed by the Artc2.1-mediated ADP-ribosylation of two immunoglobulin G (IgG) receptors, FcγR1 and FcγR2B. The modification of FcγR1 and FcγR2B diminishes its ability to bind to IgG and leads to the inhibition of IgG-mediated phagocytosis (Rissiek et al., 2017). Additionally, a variety of Artc2-targeted proteins have been discovered at the surface of immune cells (Lischke et al., 2013; Teege et al., 2015; Rissiek et al., 2017). For example, CD25 is the α-chain of the Interleukin-2(IL-2) receptor that is constitutively expressed on Tregs (Sakaguchi et al., 1995; Baecher-Allan et al., 2001) and P2X7 is an ionotropic purinergic receptor that can be expressed on immune cells. While Artc2.2 MARylates CD25 at the Arg35 and tunes the IL-2 signaling, an important signaling pathway in immune response (Teege et al., 2015). Artc2.2-mediated modification of P2X7 stimulates the death of naïve T cell death (Adriouch et al., 2001; Adriouch et al., 2007).

## 3 Readers of ADP-ribosylation

Reading the ADP-ribosylation signal by ADPr-binding proteins constitutes a major aspect of ADP-ribosylation biology. Indeed, non-covalent interactions with ADPr have profound effects on protein re-distribution and function thereby modulating cellular processes like DNA repair, DNA transcription, cell cycle and cell death (Pleschke et al., 2000; Timinszky et al., 2009; Kliza et al., 2021). Given the large number and diverse functions of proteins that respond to ADP-ribosylation signaling, a unified concept may help us to understand this growing complexity. Over the past decade, several classes of motifs, domains and modules that can bind to different forms of ADP-ribose have been identified in ADP-ribosylation readers. Among them, four classes of ADPr-binding modules have been well-characterized and will be detailed below (Table 1): PAR-binding motifs (PBMs), Macrodomains, WWE domains and PAR-binding zinc finger (PBZ) domain (Ahel et al., 2008; Kalisch et al., 2012; Barkauskaite et al., 2013b; Gupte et al., 2017). Other less studied ADP-ribosylation reader modules such as Forkhead-associated (FHA), BRCA1 C-terminus-like motif (BRCT), RNA recognition modules (RRMs) and so on (Teloni and Altmeyer, 2016; Gupte et al., 2017) will not be discussed here.

**TABLE 1:** Summary of ADP-ribosylation readers.

Reader module	Examples	Main functions	References
PBM	Histone H2A, Histone H2B, Histone H3, Histone H4, P21, p53, XRCC1, DNA-PK(CS), Ku70, DNA ligaseIII, MSH6, polymerase epsilon, DNMT1, AIF, hnRNP A1	DNA repair, chromatin rearrangements, apoptosis, cell cycle, cell death, transcription, gene expression	Pleschke et al. (2000); Gagne et al. (2003); Ji and Tulin, (2009); Wang et al. (2011); Zampieri et al. (2012); Teloni and Altmeyer, (2016)
Macrodomain	macroH2A1.1, ALC1, PARP9, PARP14	Chromatin remodeling, DNA repair	Gottschalk et al. (2009); Timinszky et al. (2009); Forst et al. (2013); Yan et al. (2013)
WWE	PARP12, RNF146, TRIP12	Stress granules formation, protein stability	Wang et al. (2012); Catara et al. (2017); Gatti et al. (2020)
PBZ	CHK1, APLF, CHFR	Cell cycle, DNA damage response, protein stability	Rulten et al. (2008); Rulten et al. (2011); Liu et al. (2013); Min et al. (2013)

PAR-binding motifs (PBMs) were originally identified from a family of DNA checkpoints proteins. In these proteins, PBMs were found to interact with PAR and overlap with important functional domains (Pleschke et al., 2000). By targeting PBM, PAR can regulate protein localization, protein-DNA interaction as well as protein degradation (Pleschke et al., 2000). PMBs are short amino acid stretches with a conserved sequence: [HKR]-X-X-[AIQVY]-[KR]-[KR]-[AILV]-[FILPV] (Pleschke et al., 2000). Kleine and Luscher (2009) denoted that the positively charged amino acid residues within the PBM consensus might mediate its electrostatic interaction with negatively charged PAR chains. PBMs are abundant in proteins involved in DNA repair, chromatin rearrangements, cell cycle regulation and many other biological processes. It is predicted that there are more than 800 PBM-containing proteins (Gagne et al., 2008). Many of these PBM-containing proteins have other high affinity PAR-binding domains. For instance, some of the heterogeneous nuclear ribonucleoprotein (hnRNP) family proteins contain RRM besides PBM (Gagne et al., 2003; Teloni and Altmeyer, 2016) and the PBMs of X-ray repair cross-complementing gene 1 (XRCC1) are part of its BRCT domains (Li et al., 2013; Teloni and Altmeyer, 2016). Thus, it is proposed that PBMs can not bind PAR in the absence of other specific binding domains (Hou et al., 2019).

The macrodomain is a globular domain composed of 130–190 amino acids, which show high structural homology but with relatively low sequence identity in different proteins. The macrodomain was first found in virus proteins (Gorbalenya et al., 1991) and was latterly identified in histone variant MacroH2A (Pehrson and Fried, 1992). Macrodomain-containing proteins are widespread across the tree of life including eukaryotes, prokaryotes, and archaea. In humans, there are 12 proteins possessing the macrodomain, but not all of them can bind to PAR (Kustatscher et al., 2005; Neuvonen and Ahola, 2009). The delicate sequence variations within the macrodomain convert this module from having no-MAR/PAR-binding ability [such as macroH2A1.2 (Timinszky et al., 2009), macroH2A2 (Timinszky et al., 2009), GDAP2 (Neuvonen and Ahola, 2009)] to possessing high MAR/PAR binding affinity [such as macroH2A1.1 (Timinszky et al., 2009), ALC1 (Gottschalk et al., 2009), PARP9 (Yan et al., 2013), PARP14 (Forst et al., 2013)], and even to showing MAR/PAR hydrolysis activity [such as MacroD1/2 (Rosenthal et al., 2013), TARG1 (Sharifi et al., 2013), PARG (Barkauskaite et al., 2013a)]. Besides, macrodomain-containing proteins with ADP-ribosylation recognization ability have different preferences for the forms of ADP-ribose. Whereas the MacroH2A1.1 macrodomains can recognize both MAR and PAR, PARP14 macrodomains specifically bind to MAR (Gibson et al., 2017). Moreover, when the macrodomain acts as a reader domain, it is present only in multidomain proteins, thus combing signal recognition and effector domains in a single protein. The macrodomain of macroH2A1.1 senses PARP1 activation in a PAR-dependent manner and the PAR recognition of macroH2A1.1 facilitates the chromatin rearrangements (Timinszky et al., 2009). Similarly, ALC1 is recruited to DNA damage sites by associating with PARP1-generated PAR chains via macrodomain, and the engagement with PAR chains activates its ATPase and chromatin remodeling activity (Gottschalk et al., 2009; Lehmann et al., 2017). PARP9 interacts with PAR and associates with PARylated proteins in response to DNA damage stress (Yan et al., 2013), whereas PARP14 recognizes MARylated PARP10 substrates and participates in PARP10 dependent signaling pathway (Forst et al., 2013).

The WWE domain is a globular structure consisting of ∼80 amino acids, which is characterized by its conserved tryptophan (W) and glutamate (E) residues. The WWE domain has been identified in three groups of proteins. In the first group, there is only one protein, DDHD2 (Gonzalez et al., 2013), the WWE domain of which can not bind to PAR as shown in *in vitro* PAR-binding experiment (Wang et al., 2012). The other two families of WWE-containing proteins are associated with either ADP-ribosylation or ubiquitination. ARTDs, such as PARP14, PARP11, PARP12, PARP13, and PARP7, containing one or two WWE domains constitute the second group of WWE-containing proteins (detailed in the “Writers” section) (Gupte et al., 2017; Hou et al., 2019). It’s noteworthy that WWE domain, as well as macrodomain, only exists in ARTDs that do not generate PAR chains, indicating an unknown interplay between the writing and the reading of ADP-ribose. The third group of WWE-containing proteins is composed of ubiquitin ligases, including Deltex1-4, TRIP12 and RNF146 (Aravind, 2001). The ADP-ribose moiety binding preferences of the WWE domain varies in the different group of proteins. While the WWE domain of PARP11 prefers to interact with the terminal ADP-ribose (He et al., 2012), the RNF146 WWE domain specifically recognizes the iso-ADP-ribose moiety within PAR chains (He et al., 2012; Wang et al., 2012). RNF146 specifically recognizes the PARylated proteins and targets them for ubiquitin-mediated proteasomal degradation (Wang et al., 2012; Vivelo et al., 2019). Recently, Gatti et al. (2020) demonstrate that TRIP12, just like RNF146, can bind to PARylated PARP1 via its WWE domain, leading to its poly-ubiquitylation and proteasomal degradation.

PBZ is a Cys2-His2 type zinc-finger motif with PAR-binding properties, which is ∼30 amino acids long and possesses a consensus sequence of [K/R]xxCx [F/Y]GxxCxbbxxxxHxxx [F/Y]Xh (Ahel et al., 2008; Isogai et al., 2010). It specifically binds to the adjacent ADP-ribose groups and recognizes the substrates mainly through hydrogen bonds as lack of secondary structure (Eustermann et al., 2010; Isogai et al., 2010; Li et al., 2010). PBZ domain was first identified in eukaryotic DNA repair/checkpoint proteins: APLF (aprataxin PNK-like factor) and CHFR (checkpoint protein with FHA and RING domains) (Ahel et al., 2008). While CHFR contains only one PBZ, APLF possesses tandem PBZs which generate synergy in PAR binding and remarkably enhance its PAR binding affinity (Eustermann et al., 2010; Oberoi et al., 2010). Compared with other PAR-binding structures, the PBZ domain in mammals is much less widespread and is only present in very few proteins (Teloni and Altmeyer, 2016). Apart from APLF and CHFR, SNM1A (sensitive to nitrogen mustard 1 A) was reported to have a PBZ domain but its PBZ domain lacked critical PAR-binding residues and was predicted not to bind PAR (Ahel et al., 2008; Oberoi et al., 2010; Barkauskaite et al., 2013b). Moreover, a variant PBZ motif was found in CHK1 and mutation of this motif abolished its PAR-binding ability (Min et al., 2013). PAR recognition is closely linked to the cellular function of PBZ-containing proteins. APLF is a purinic-apyrimidinic (AP) endonuclease. By binding to PAR with PBZ domains, APLF is recruited to DNA damage sites and promotes DNA repair (Rulten et al., 2008; Rulten et al., 2011; Chen et al., 2018). Similarly, the recruitment of CHFR (a ubiquitin E3 ligase) to DNA double-strand breaks (DSBs) is PAR-dependent. At DSBs, CHFR binds to and ubiquitinates the auto-PARylated PARP1, thereby facilitating its eviction from chromatin and promoting its degradation following DNA damage. Depletion of *Chfr* prolongs the retention of PARP1 at the DNA damage sites (Liu et al., 2013). CHK1 is another key mediator of DNA damage response. It interacts with PAR chains via the variant PBZ domain. PAR recognition is required for its efficient retention and full activation at damaged replication forks, which is essential for blocking replication fork progression and promoting DNA repair (Min et al., 2013).

## 4 Erasers of ADP-ribosylation

ADP-ribosylation plays an important role in various biological processes and is tightly controlled both spatially and temporally. Erasers that can terminate the ADP-ribosylation modification (Rack et al., 2020) are important regulators of ADP-ribosylation signaling. The erasers can be broadly categorized into three distinct families: the macrodomain-containing enzymes, the ADP-ribosyl-acceptor hydrolases (ARHs) and some enzymes with phosphodiesterase activity that are recently found to process the reversal of ADP-ribosylation (Daniels et al., 2015; Palazzo et al., 2015; Palazzo et al., 2016). Next, we will provide an outline of these ADP-ribose erasers (Table 2).

**TABLE 2:** Summary of ADP-ribosylation erasers.

Eraser name	Classification	Protein substrates	References
PARG	Macrodomain	PAR-protein	Slade et al. (2011); Barkauskaite et al. (2013a)
TARG1	Macrodomain	PAR-Glu MAR-Asp/Glu	Rosenthal et al. (2013); Sharifi et al. (2013)
MacroD1	Macrodomain	MAR-Asp/Glu	Rosenthal et al. (2013)
MacroD2	Macrodomain	MAR-Asp/Glu	Rosenthal et al. (2013)
ARH1	ARH family	MAR-Arg	Oka et al. (2006); Kato et al. (2007)
ARH3	ARH family	PAR-protein MAR-Ser	Mueller-Dieckmann et al. (2006); Oka et al. (2006)
NUDT9	Nudix family	PAR-protein	Palazzo et al. (2015)
NUDT16	Nudix family	PAR-protein MAR-protein	Palazzo et al. (2015)
ENPP1	ENPP family	PAR-protein MAR-protein	Palazzo et al. (2016)

Macrodomain is a widespread, conserved protein fold, which can be found in “writers”, “readers” and “erasers” of ADP-ribosylation (Rack et al., 2016). There are four well-characterized macrodomain-containing proteins (PARG, TARG1, MacroD1 and MacroD2) that possess hydrolase activity (Feijs et al., 2013a; Rack et al., 2016). It’s worth noting since the amino acid sequence of PARG bears no resemblance to other macrodomain-containing proteins, the macrodomain fold in PARG is not deduced from sequence alignments, but is identified by structure analysis (Slade et al., 2011). PARG specifically hydrolyzes the glycosidic bonds between ADP-ribose units and degrades the PAR chains in an exo- or endo-glycosidic manner (Barkauskaite et al., 2013a; Rack et al., 2021). Extensive research reveals that it mainly acts as an exoglycosidase due to its higher affinity to the end of PAR chains (Barkauskaite et al., 2013a). Although PARG is an efficient dePARylation enzyme and accounts for ∼90% PAR catabolism, it is unable to remove the protein-bound proximal ADP-ribose (Slade et al., 2011; Barkauskaite et al., 2013a), the degradation of which instead is performed by MAR-erasers. TARG1, MacroD1 and MacroD2 are such MAR-erasers that possess deMARylation activity and specifically cleave the ester bond between terminal ADP-ribose and the aspartate and glutamate residues (Rosenthal et al., 2013; O'Sullivan et al., 2019). Of note, TARG1 can not only reverse MARylated protein but also remove the entire PAR chains from the glutamate residue on the PARylated proteins (Sharifi et al., 2013; O'Sullivan et al., 2019).

The ARH family consists of three members, ARH1-3. In humans, the ARH1 shows 47%, 22% sequence identity with ARH2 and ARH3, respectively (Oka et al., 2006; Kato et al., 2015). Although sharing substantial sequence similarity, ARH1-3 exhibit significant diverse enzymatic activities (Bu et al., 2019). ARH1 is a MAR-eraser and specifically hydrolyzes the N-glycosidic bond of MARylation modified arginine residues (Oka et al., 2006), whereas ARH3 possesses both dePARylation and deMARylation activity, which preferentially cleaves the O-glycosidic bond of PAR chains and MARylation modified serine residues (Oka et al., 2006; Fontana et al., 2017). While ARH1 also displays a hydrolytic activity towards the O-glycosidic bond in PAR chains and O-acetyl-ADP-ribose (OAADPr), its activity is much weaker than that of ARH3 (Oka et al., 2006; Ono et al., 2006; Kasamatsu et al., 2011; Mashimo et al., 2014; Ishiwata-Endo et al., 2020). Furthermore, the O-glycosidic bond between ADP and DNA/RNA can be hydrolyzed by ARH3, but not ARH1 (Moss et al., 1985; Rack et al., 2018; Munnur et al., 2019; Ishiwata-Endo et al., 2020; Weixler et al., 2021). Although both ARH1 and ARH3 have been identified to possess hydrolase activity, no enzyme activity has been observed in ARH2 (Oka et al., 2006; Ono et al., 2006).

Several pyrophosphatases have been found to participate in ADP-ribose metabolism. These include two unrelated classes of proteins: the nucleoside diphosphate linked to a variable moiety X (Nudix) (McLennan, 2006; Daniels et al., 2015; Palazzo et al., 2015; Kulikova and Nikiforov, 2020) and ectonucleotide pyrophosphatase (ENPP) (Palazzo et al., 2016). Rather than completely removing the ADP-ribose moiety, these pyrophosphatases digest the phosphodiester bond which links the adenosine to the ribose moiety in ADP-ribose, liberating the AMP or phosphoribose-AMP and leaving phosphoribose remnants attached to the proteins. The physiological function associated with these phosphoribose remnants remains unclear, but the pathological accumulation of glutamyl ribose 5-phosphate has been observed in patients with neurologic degeneration and renal failure (Williams et al., 1984). Despite having a similar catalytic mechanism, pyrophosphatases have different substrate specificities. Moreover, as they target the phosphodiester bond, their substrates have no amino-acid specificity (Daniels et al., 2015; Palazzo et al., 2015; Palazzo et al., 2016). For Nudix family members, only NUDT9 and NUDT16 have been shown to hydrolyze protein-conjugated ADP-ribose (O'Sullivan et al., 2019; Kulikova and Nikiforov, 2020). NUDT16 is the only known Nudix member that can digest both MARylated and PARylated proteins (Palazzo et al., 2015) and has a broad target spectrum including PAR-protein, PAR-DNA, MAR-protein, MAR-DNA, MAR-RNA, free ADP-ribose and so on (Palazzo et al., 2015; Talhaoui et al., 2016; Munnur and Ahel, 2017; Munnur et al., 2019; Zhang F. et al., 2020; Kulikova and Nikiforov, 2020). NUDT9 possesses phosphodiesterase activity against PARylated proteins, but its activity is much lower than observed in NUDT16 (Palazzo et al., 2015). In addition to protein-conjugated PAR, free ADP-ribose and OAADPr are also substrates of NUTD9 (Perraud et al., 2003). Mammalian ENPP1 is a recently characterized non-Nudix pyrophosphatases that digest both PAR and MAR from acceptor proteins like NUDT16 (Palazzo et al., 2016). Within the ENPP protein family, Snake Venom Phosphodiesterase exhibits the same phosphodiesterase activity as mammalian ENPP1 dose (Oka et al., 1978).

## 5 Discussion

ADP-ribosylation is one of the most important PTMs that has been studied for over 50 years. As a reversible PTM, the life cycle of ADP-ribosylation includes deposition, recognition, and degradation of ADP-ribose on its substrates, which is mediated by dedicated enzymes known as writers, readers, and erasers, respectively.

ARTs are the main writers of ADP-ribosylation and 23 ARTs members have been discovered in mammals (Luscher et al., 2021). Although the recent research has substantially expanded our knowledge about ARTs’ contribution to a variety of different fields beyond their traditional roles (Leung et al., 2011; Meyer-Ficca et al., 2015; Zhang et al., 2015; Huang et al., 2016; Gozgit et al., 2021; Xing et al., 2021; Grunewald et al., 2019; Iwata et al., 2016; Tang et al., 2018), the understanding of the broad spectrum of ARTs-dependent biological processes is still in its infancy and many questions remain unanswered: First, some ARTs, such as PARP4, PARP8, PARP15, Artc3, Artc4, Artc5, are still poorly understood and a better characterization of the structure and function of these ART members is required. In addition, there are various ARTs and their potential targets in cells, so in a given stimulation, the molecular basis for the activation of specific ARTs and the downstream effectors of this ART need to be addressed. However, the identification of ARTs’ activators and targets faces many difficulties as the related technologies have not yet reached maturity. Besides, identification of ADP conjunction sites at targets is an essential step to understanding the catalytic-dependent biological roles of ARTs, but a full list of ART modification sites has not been established. Furthermore, the biological relevance of site-specific ADP-ribosylation events remains largely unknown despite it has been demonstrated that serine ADP-ribosylation is the most form of ADPr in DNA damage signaling (Palazzo et al., 2018).

ADP-ribosylation readers are pivotal in the transduction of ADP-ribosylation signals. Several structural domains and modules have been identified to recognize ADP-ribosylation and convey the information downstream (Pleschke et al., 2000; Karras et al., 2005; Ahel et al., 2008; Wang et al., 2012; Dasovich et al., 2021; Kliza et al., 2021), yet we know relatively little about the modulatory effect of many ADP-ribose readers. Moreover, it appears that different reader modules recognize different sites within MAR/PAR (Timinszky et al., 2009; Isogai et al., 2010; He et al., 2012), probably implying distinct biological outcomes, whereas it is also observed that the same cellular process can be performed by various reader modules (Pleschke et al., 2000; Rulten et al., 2008; Timinszky et al., 2009; Rulten et al., 2011; Liu et al., 2013; Gatti et al., 2020). Thus, this raises the question: What determines which reader is needed in the cellular process where they can all function? How do reader modules distinguish between different target proteins *in vivo*? Currently, it is proposed that the structural heterogeneity of PAR can influence the binding characteristics of its readers and affect the biological outcomes (Krietsch et al., 2012; Chen et al., 2018; Moor et al., 2020; Dasovich et al., 2021; Reber and Mangerich, 2021), so there should be more ADP-ribosylation readers still to be discovered due to the complexity of PAR structure.

Whilst substantial progress has been made in revealing the machinery responsible for ADP-ribosylation reversal (Barkauskaite et al., 2013a; Rosenthal et al., 2013; Fontana et al., 2017; Rack et al., 2021), very little is known about how the ADP-ribosylation erasers are regulated. Moreover, the linkage selectivity of the ADP-ribosylation erasers is largely unresolved, and particularly, the mechanisms of site-specific MAR-erasers have been understudied and need further detailed analysis. Besides, elucidating the interplay between ADP-ribosylation writers, readers and erasers at the molecular and cellular level is necessary, which may help in understanding the ADP-ribosylation associated physiological and pathological processes.
